# Thyroid disorders, thyroid autoimmunity, and anxiety—a systematic review and meta-analysis of population-based studies

**DOI:** 10.7717/peerj.21446

**Published:** 2026-08-04

**Authors:** Beatrice Ivens, Henry Bode, Tom Bschor, Jonathan Henssler, Christopher Baethge

**Affiliations:** 1 Department of Psychiatry and Psychotherapy, Universität Köln, Cologne, Germany; 2 Department for Psychiatry and Psychotherapy, Technische Universität Dresden, Dresden, Germany; 3 Department of Psychiatry and Psychotherapy, Charité–Universitätsmedizin Berlin, Corporate Member of Freie Universität Berlin and Humboldt-Universität zu Berlin, St Hedwig Hospital, Humboldt Universität Berlin, Berlin, Germany

**Keywords:** Population-based, Systematic review, Meta-analysis, Hypothyroidism, Hyperthyroidism, Anxiety disorders, Autoimmunity

## Abstract

**Objectives:**

Previous studies, largely based on case–control samples, have suggested that hypothyroidism, hyperthyroidism, and thyroid autoimmunity may be associated with an increased risk of anxiety. However, whether a true association exists remains unclear, as population-based studies, which draw on more representative samples and are therefore less prone to selection bias, have yielded conflicting results. This study aimed to investigate the association between thyroid dysfunction and anxiety disorders in population-based cohort and cross-sectional studies.

**Methods:**

We conducted a systematic literature search in PubMed, PsycINFO, and Embase databases from inception to March 2026. Two reviewers independently selected population-based cohort and cross-sectional studies reporting laboratory- or International Classification of Diseases (ICD)-based diagnoses of thyroid dysfunction or autoimmunity, and anxiety outcomes according to validated criteria or rating scales. Study quality was assessed using the Newcastle-Ottawa Scale. All meta-analyses were conducted using random-effects models, and heterogeneity was quantified using the *I*^2^ statistic (pre-registration in Prospero: CRD42020164791)

**Results:**

Out of 2,548 screened articles, 12 studies comprising 676,658 participants were included. Six studies dealt with hypothyroidism, seven with hyperthyroidism, and four with autoimmunity. Meta-analyses revealed small and mostly statistically non-significant positive summary estimates for hypothyroidism (OR 1.13, 95% CI [0.77–1.65]; *I*^2^ = 86%), hyperthyroidism (OR 1.38, 95% CI [1.03–1.83]; *I*^2^ = 86%), and thyroid peroxidase antibody (TPOAb) (OR 1.47, 95% CI [0.73–2.94]; *I*^2^ = 65%). Sensitivity and subgroup analyses, as well as studies summarized qualitatively, showed patterns consistent with the main results.

**Limitations:**

The number of studies is small, and thyroid pathology as well as anxiety are heterogeneously defined.

**Conclusion:**

The evidence is inconclusive: Small positive associations seem likely, but confidence intervals are also in agreement with intermediate positive and even small protective effects, suggesting either limited data or the absence of relevant associations between thyroid pathology and anxiety. Further population-based studies based on established diagnostic criteria are warranted.

## Introduction

The global prevalence of hypothyroidism is considered to be 1–2% (Vanderpump, 2011). It is most frequently caused by autoimmune disease, *i.e.*, Hashimoto thyroiditis (Dayan & Daniels, 1996), and affects more women than men (Hu et al., 2022). Hyperthyroidism has a similar female preponderance, with prevalences of 2–3% and 0.2–0.3% among women and men, respectively (Madariaga et al., 2014; Hollowell et al., 2002). It is also primarily the result of autoimmunity, in this case, Graves’ disease (De Leo, Lee & Braverman, 2016).

Anxiety disorders have a prevalence of 14% and are thus the most common psychiatric conditions (Wittchen et al., 2011). Both anxiety disorders and thyroid dysfunction, particularly hyperthyroidism, may present with similar symptoms, such as nervousness, insomnia, and impaired concentration (De Leo, Lee & Braverman, 2016; Aristotle, Georgette & Mani, 2020). Further, physical signs, *e.g.*, palpitations, sweating and diarrhea are found in both diseases (Mattesi et al., 2022; Showraki, Showraki & Brown, 2020; Rickels & Rynn, 2001). There is also evidence that thyroid hormones affect brain monoamine metabolism (Chanoine et al., 1992; Visser et al., 2008; Williams, 2008; Bauer, Heinz & Whybrow, 2002) which is thought to be altered in anxiety disorders, too (Eison, 1990).

Symptom overlap and similar brain metabolism pathways suggest a relationship between both groups of disorders and beg the question of whether people diagnosed with thyroid disorders are at a higher risk of developing anxiety disorders.

A meta-analysis by Siegmann et al. (2018), reported an odds ratio of 2.3 for anxiety disorders among patients diagnosed with hypothyroidism. The magnitude of this odds ratio can be questioned, as predominantly small case-control studies were used to determine the effect size. Such samples often originate with specialty clinics, be it in endocrinology or psychiatry departments, and thus are especially prone to selection bias as they might be enriched for particularly severe cases. Further, the observed prevalence or incidence of anxiety disorders in individuals with hypo- or hyperthyroidism varies widely depending on the underlying study type and population (Baethge, 2018). Population- based studies, by design, provide better insights into general disease risks and comorbidity. A population-based cohort study with 30,500 participants even suggested a negative association between subclinical hypo- and hyperthyroidism and anxiety symptoms (Engum et al., 2002). On the other hand, another study with about 2,000 participants revealed an association between diagnosed hypothyroidism and the development of anxiety disorders (Ittermann et al., 2015).

Therefore, this study aims to determine whether an association between thyroid dysfunction and anxiety disorders exists. To avoid bias related to case-control studies, only population-based cohort and cross-sectional studies were considered, applying a systematic method and aiming to include all suitable studies.

## Method

This systematic review and meta-analysis sought to determine whether there is an association between thyroid disease and anxiety disorders in the general population. The protocol for this systematic review was submitted to PROSPERO in January 2020 and registered *a priori* in April 2020 (CRD42020164791) as part of a larger project on the co-occurrence of thyroid disorders with depression and anxiety, parts of which have been published elsewhere (Bode et al., 2022; Bode et al., 2021). Because delays in the registration process were expected, formal screening of search results against the eligibility criteria started shortly after submission but before the registration was officially published, in order to maintain project timelines.

In conducting this study, we adhered to the Cochrane Collaboration Handbook for Systematic Reviews, and in its presentation, we followed the PRISMA Guidelines (Page et al., 2021).

The main stages of the present study–screening of the literature, evaluating full-texts for inclusion, data extraction, and risk-of-bias ascertainment–were carried out independently by two researchers (BI and HB). Controversies were solved through discussion among the authors (BI, HB, CB).

## Literature Search

We systematically searched MEDLINE, EMBASE, and EBSCOhost (PsycINFO, PSYNDEX, PsycArticles) from database inception until March 29, 2026. Search strings were developed *a priori* using Boolean operators to combine terms for thyroid dysfunction (*e.g.*, ‘Hashimoto’, ‘hypothyroid’, ‘Graves’ Disease*’, ‘hyperthyroid’) with terms for anxiety (*e.g.*, ‘anxiety*’, ‘fear’, ‘panic’) and were adapted to the syntax requirements of each database. The strategy was refined through repeated testing to ensure high sensitivity, and full search strings are provided in the Supplement. Apart from the exclusion of animal studies, no studies were actively excluded from the literature search (including grey literature). No date restrictions were applied. In addition, we manually screened bibliographies of included articles and reviews that surfaced during the literature search. In a Web of Science forward search, we also screened citations to the articles eventually included.

### Study design

We focused on population-based cohort and cross-sectional studies because they provide more generalizable estimates and avoid the strong selection biases typical of case–control designs. Previous research on thyroid dysfunction and anxiety has relied on clinically recruited case–control samples, which often report inflated effect sizes due to the inclusion of patients with more severe presentations and non-representative controls, whereas population-based studies have yielded smaller or even negative associations. To capture studies with near-population representativeness, we also included quasi–population-based designs, such as studies recruiting through general practitioners in the United Kingdom (UK), where almost all residents are registered with a general practitioner (GP). Population-based sampling frames better reflect real-world prevalence and comorbidity patterns and therefore offer a more reliable basis for evaluating associations in the general population. For these reasons, case–control studies were excluded.

### Exposure

We included studies on conditions typically impairing thyroid function: hypothyroidism, hyperthyroidism, and autoimmune diseases (Hashimoto thyroiditis, Graves’ disease). Case definitions were based on laboratory measurements of TSH, peripheral thyroid hormones, such as FT3/fT4, thyroid peroxidase (TPO), medical records, or self-reported. We distinguished between hypo- and hyperthyroidism, and between subclinical and overt thyroid disorder. Subclinical hypo- or hyperthyroidism is defined as a laboratory diagnosis in which only TSH levels are abnormally elevated or decreased. For the diagnosis of an overt disorder, in addition to TSH abnormalities, peripheral thyroid hormone values (*e.g.*, fT3/fT4) needed to be decreased or increased beyond laboratory references (as defined by local laboratory reference values in a given study). These definitions follow internationally accepted clinical and laboratory criteria for thyroid dysfunction and were chosen to ensure valid and comparable exposure classification across studies. Using standardized laboratory parameters reflects current clinical guidelines and allows a clear distinction between subclinical and overt disorders. Autoimmune thyroiditis and Graves’ disease were approximated using thyroid peroxidase antibodies (TPOAb) and TSH receptor antibodies (TRAb) positivity, respectively, recognizing that antibody status does not constitute a clinical diagnosis and serves only as an epidemiological proxy for underlying autoimmune activity. Including both functional (hypo- and hyperthyroidism) and autoimmune conditions provides a comprehensive framework that captures the major pathophysiological mechanisms potentially linked to anxiety outcomes.

### Outcome

Outcomes included frequency figures for anxiety disorders, defined as a diagnosis of any anxiety or specific anxiety disorder according to established diagnostic systems (e.g., ICD-10, DSM-IV), an above-threshold score on a validated rating scale for anxiety disorders such as the HADS-A (Zigmond & Snaith, 1983), or a diagnosis based on self-report or extraction from medical records. If more than one anxiety disorder was reported, we first prioritized summarizing data (“any anxiety disorder”) then generalized anxiety disorder (GAD).

### Exclusion criteria

We excluded case–control studies and other clinically recruited or non–population- based samples, studies lacking valid definitions of thyroid dysfunction or anxiety outcomes, and studies specifically focusing on pregnant and postpartum women as differences in the interplay between thyroid disorders and anxiety in these populations cannot be excluded (Gaberšček & Zaletel, 2011; Medici et al., 2015).

### Risk of bias

Risk of bias was assessed using the Newcastle-Ottawa Scale (NOS) with adaptations for cohort and cross-sectional studies (Wells et al., 2000).

## Data Synthesis and Analysis

### Primary outcome and statistical analysis

The occurrence of anxiety disorders in individuals with *versus* without hypothyroidism or hyperthyroidism (primary outcomes) or autoimmunity was compared, separately for each thyroid pathology. Random-effects meta-analyses were conducted to estimate pooled odds ratios (ORs) with 95% confidence intervals (CIs). An odds ratio > 1 indicates increased odds of anxiety disorders, <1 indicates decreased odds (Szumilas, 2010). Heterogeneity between studies was assessed using the *I*^2^ statistic, and 95% prediction intervals were calculated where applicable. All analyses were carried out in Comprehensive Meta-Analysis (CMA), Version 2, and metaHUN (Umaroglu & Ozdemir, 2018) using DerSimonian and Laird’s tau^2^ estimation and Wald 95% confidence intervals. For co-primary outcomes, we conducted sensitivity analyses based on Paule-Mandel tau^2^ estimators and Knapp-Hartung adjustments for calculating 95% confidence intervals.

### Subgroup analyses

Subgroup analyses were conducted to distinguish between subclinical and overt thyroid dysfunction.

### Reporting bias

An analysis of small study effects based on Egger’s test and trim-and-fill-analyses was planned.

## Results

**Figure 1:**
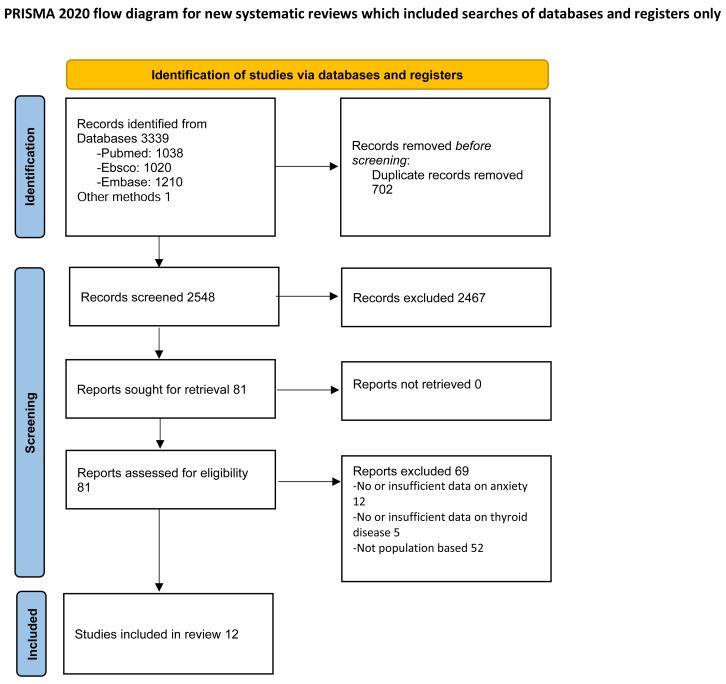
PRISMA flowchart of the study selection process PRISMA guidelines (Page et al., 2021).

We screened a total of 2,548 articles, out of which 81 were evaluated at full-text level and twelve included in our analysis (Fig. 1). The articles were published between 2002 (Engum et al., 2002) and 2025 (Lee et al., 2025). Enrolment varied from 130 (Shrestha et al., 2016) to 497,726 (Soheili-Nezhad et al., 2023), with a total of 676,658 subjects. Studies included patients across an age range between 18 and above 90 years (see Table 1 for study characteristics).

**Table 1:** Study characteristics.

Source	Study type	No. of participants	Sex	Age [y]^1^	Thyroid disorder	Assessment of thyroid disorder	Assessment of anxiety	NOS score
Benseñor et al. (2016)	Cross-sectional	13,414	Mixed	51.7	Subclinical hypo- and hyperthyroidism	FT4, TSH	Clinical diagnosis (GAD, PD)	7
Carta et al. (2004)	Cross-sectional	222	Mixed, female, male	>18	TPO antibodies	TPO-abs	Clinical diagnosis (based on DSM-IV generalized anxiety disorder, panic disorder)	7
Engum et al. (2002)	Cross-sectional	30,589	Mixed	40–89	Overt and subclinical hypo- and hyperthyroidism,	FT4, TSH	HADS-Anxiety (Cut off 8 points)	9
Engum et al. (2005)	Cross-sectional	2,445	Mixed	40–84	TPO antibodies	FT4, TSH	HADS-Anxiety (Cut off 8 points)	9
Ittermann et al. (2015)	Cohort	2,142	Mixed	20–79	Overt hypo- and hyperthyroidism, TPO antibodies	TSH, TPO-abs	Clinical diagnosis (based on DSM-IV Anxiety excl. Phobias)	8
Lee et al. (2023)	Cross-sectional	398	Mixed	*ϕ*64	Diagnosis of Thyroid Eye disease (TED)	Medical record, self-reported	Medical record, self-reported	7
Lee et al. (2025)	Cohort	20,851	Mixed	*ϕ*44.6	Hyperthyroidism	ICD-Diagnosis from Medical record	ICD-Diagnosis from Medical record	9
Panicker et al. (2009)	Cross-sectional	27,013	Female, Male	NA	Serum TSH levels	TSH	HADS-Anxiety (Cut off 8 points)	9
Roberts et al. (2006)	Cross-sectional	5,868	Mixed	65–98 *ϕ*73	Overt and subclinical hypo- and hyperthyroidism	FT4, TSH	HADS-Anxiety	8
Samuels et al. (2016)	Cross-sectional	539	Male	≥65	Thyroid function within reference range	FT4, TSH	Goldberg Anxiety Scale (cut off 4 points)	7
Shrestha et al. (2016)	Cross-sectional	130	Mixed	58–68 *ϕ*63.12	Serum TSH and FT4 levels	FT4, TSH	State-trait anxiety inventory	7
Soheili-Nezhad et al. (2023)	Cross-sectional	497,726	Mixed	40–72 *ϕ*56.6	Overt hypo- and hyperthyroidism	Medical record, self-reported	Self-reported diagnoses	8

**Notes.**

The Hospital Anxiety and Depression Scale (HADS) is a self-reporting scale consisting of 7 items for anxiety. Each item is scored from 0 to 3. A threshold value exists from 8 points for the presence of a mild, 11 for moderate and 15 for a severe anxiety disorder (Zigmond & Snaith, 1983). The Goldberg Anxiety Scale is a scale consisting of nine questions, which are answered with yes and no. There is a threshold value of 4 points above which it can be assumed that clinically relevant anxiety symptoms are present (Baxter et al., 2013).

The State-Trait Anxiety Inventory is a questionnaire consisting of 20 questions. Respondents answer each item on a 4-point scale ranging from 1 to 4, with a total score ranging from 20 to 80. A cut-off score of 40 is typically used to indicate probable clinical anxiety (Baxter et al., 2014). In Carta et al. we selected GAD figures over results for all anxiety disorders because OR and 95% CIs of the latter seemed erroneous. In a sensitivity analysis, we tentatively re-calculated an OR from the results presented elsewhere in the paper and arrived at a slightly higher OR for the entirety of anxiety disorders relative to GAD (3.4 instead of 2.7) that would have moved the summary estimate for hypothyroidism and anxiety disorders from 1.13 ([0.77–1.65], see Table 2) to 1.22 ([0.82–1.83], I-squared: 88%, *n* = 5 studies).

1 In this table, age is presented as provided in the original studies.

Thyroid function was evaluated in nine studies through laboratory parameters determined by immunoassays. Three studies used diagnoses from electronic health records and self-reported diagnoses (Lee et al., 2025; Soheili-Nezhad et al., 2023; Lee et al., 2023). Anxiety disorders were identified in one study using self-reported anxiety diagnoses (Soheili-Nezhad et al., 2023) Two others utilized electronic health records and standardized self-report questionnaires (Lee et al., 2025; Lee et al., 2023). In three studies (Ittermann et al., 2015; Benseñor et al., 2016; Carta et al., 2004), diagnoses were obtained through structured, standardized interviews based on ICD-10 (CIS-R, CIDIS, M-CIDI). One study (Samuels et al., 2016) used the Goldberg Anxiety Scale (GAS), four (Engum et al., 2002; Engum et al., 2005; Panicker et al., 2009; Roberts et al., 2006) the Hospital Anxiety and Depression Scale (HADS-A) and one study (Shrestha et al., 2016) used the State and Trait Anxiety Inventory (STAI).

Six studies investigated hypothyroidism: two were restricted to overt hypothyroidism (Ittermann et al., 2015; Soheili-Nezhad et al., 2023), two examined both overt and subclinical hypothyroidism (Engum et al., 2002; Roberts et al., 2006), and one only the subclinical form (Benseñor et al., 2016).

Seven studies regarding hyperthyroidism were included, with three studies (Ittermann et al., 2015; Lee et al., 2025; Soheili-Nezhad et al., 2023) examining only overt hyperthyroidism, two exploring both subclinical and overt hyperthyroidism (Engum et al., 2002; Roberts et al., 2006), and one (Benseñor et al., 2016) investigating subclinical forms only. One study (Lee et al., 2023) focused on thyroid eye disease (TED), a condition most commonly associated with Graves’ disease and typically caused by TSH receptor antibodies (TRAb) (Burch et al., 2022).

Four studies examined thyroid autoimmunity, three regarding the presence of thyroid peroxidase antibodies (TPOAb) (Ittermann et al., 2015; Carta et al., 2004; Engum et al., 2005).

The pool of included population-based studies comprised ten cross-sectional and two cohort studies (Ittermann et al., 2015; Lee et al., 2025). The study by Benseñor et al. (2016) focused on civil servants, who are not representative of the entire Brazilian population. However, we did not identify any relevant increased risk of bias concerning our research question. The population from the study by Roberts et al. (2006) was drawn from general practices. Nevertheless, it should be noted that in England, almost every resident is registered with a GP.

### Risk of bias

Five studies (Engum et al., 2002; Ittermann et al., 2015; Lee et al., 2025; Engum et al., 2005; Panicker et al., 2009) had a low risk of bias. These studies achieved all or all but one points in the Newcastle-Ottawa Scale (NOS) rating system, and no study received less than seven points on the NOS (see Table 1 for scores on the NOS).

### Hypothyroidism

**Figure 2:**
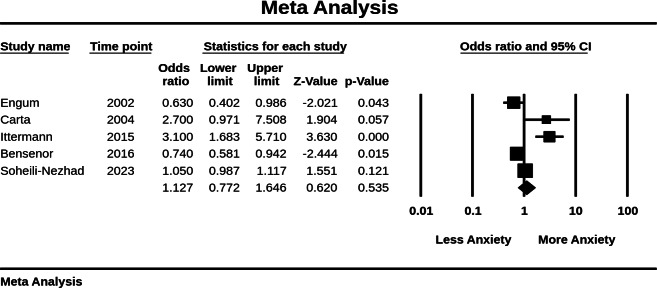
Forest plot: random effects meta-analysis of hypothyroidism and clinical anxiety in population-based studies.

Across all studies (*n* = 5), the odds ratio was 1.13 (95% CI [0.77–1.65]), with high heterogeneity (*I*^2^ = 86%). Subgroup analyses did not yield statistically significant findings: For overt hypothyroidism, the OR was 1.21 (95% CI [0.65–2.27]), and among patients with subclinical hypothyroidism the OR was similar: OR 0.90 (95% CI [0.58–1.40]) (Table 2, Fig. 2).

**Table 2:** Random effects meta-analyses on associations of thyroid and anxiety disorders in population-based studies.

	**RE-MA** **calculation**	**Studies**	**Summary Odds ratio** **(95% CI)**	**Prediction interval**	**I-square**
**Hypothyroidism**					
all (main analysis)	DL & Wald	5	1.127 [0.772–1.646]	0.31–4.14	86%
all	PM & Knapp-Hartung	5	1.229 [0.506–2.985]	0.10–15.8	95%
clinical only	DL & Wald	3	1.210 [0.647–2.265]	n.a.	88%
subclinical only	DL & Wald	2	0.898 [0.576–1.399]	n.a.	70%
**Autoimmunity:** TPO-Abs only	DL & Wald	3	1.465 [0.731–2.938]	n.a.	65%
**Hyperthyroidism**					
all (main analysis)	DL & Wald	6	1.375 [1.032–1.833]	0.54 –3.52	86%
all	PM & Knapp-Hartung	6	1.354 [0.859–2.135]	0.39 –4.71	91%
w/o Lee, 2023	DL & Wald	5	1.196 [0.942–1.517]	0.43–3.36	72%
clinical only	DL & Wald	4	1.255 [0.981–1.604]	0.47–3.37	72%
subclinical only	DL & Wald	2	1.085 [0.860–1.369]	n.a.	0%
**autoimmunity:TRAbs** only	n.a.	1 no MA	2.82 [2.26–3.70]	n.a.	n.a.

**Notes.**

Main analyses (a) sensitivity analyses utilizing Paule-Mandel tau-square estimators and Knapp-Hartung adjustments, as well as (b) different subgroup analyses. RE-MA, random-effects meta-analysis; DL, DerSimonian & Laird tau square estimator; PM, Paule-Mandel tau square estimator; 95% CI, 95-percent confidence interval; Wald, 95% CI according to Wald; Knapp-Hartung, 95% CI calculated according to Knapp and Hartung; Clinical: abnormal TSH with fT3/fT4 outside the reference range; Subclinical: abnormal TSH with normal fT3/fT4; TPO-Abs, Thyroid peroxidase antibodies; TRAb, thyroid-stimulating hormone (TSH) receptor antibodies; n.a., not applicable; w/o Lee, 2023: Excluded because the TED cohort is highly selected and prone to bias.

We did not include the study by Roberts et al. (2006) in our meta-analysis due to the absence of dichotomous outcome data. In a cohort of 5865 individuals recruited from a primary care register, participants with subclinical hypothyroidism had higher scores on the HADS-A (Mean: 4.98 [95% CI [4.43–5.53]) compared to patients with overt hypothyroidism (4.20 [3.17–5.22]) or euthyroidism (4.8 [4.62–5.03]). In all groups, the upper limits of the 95%-confidence intervals remained well below the cut-off of 8 points for clinically relevant symptomatic anxiety, and thus the differences have no bearing on our focus on clinical anxiety.

### Hyperthyroidism

**Figure 3:**
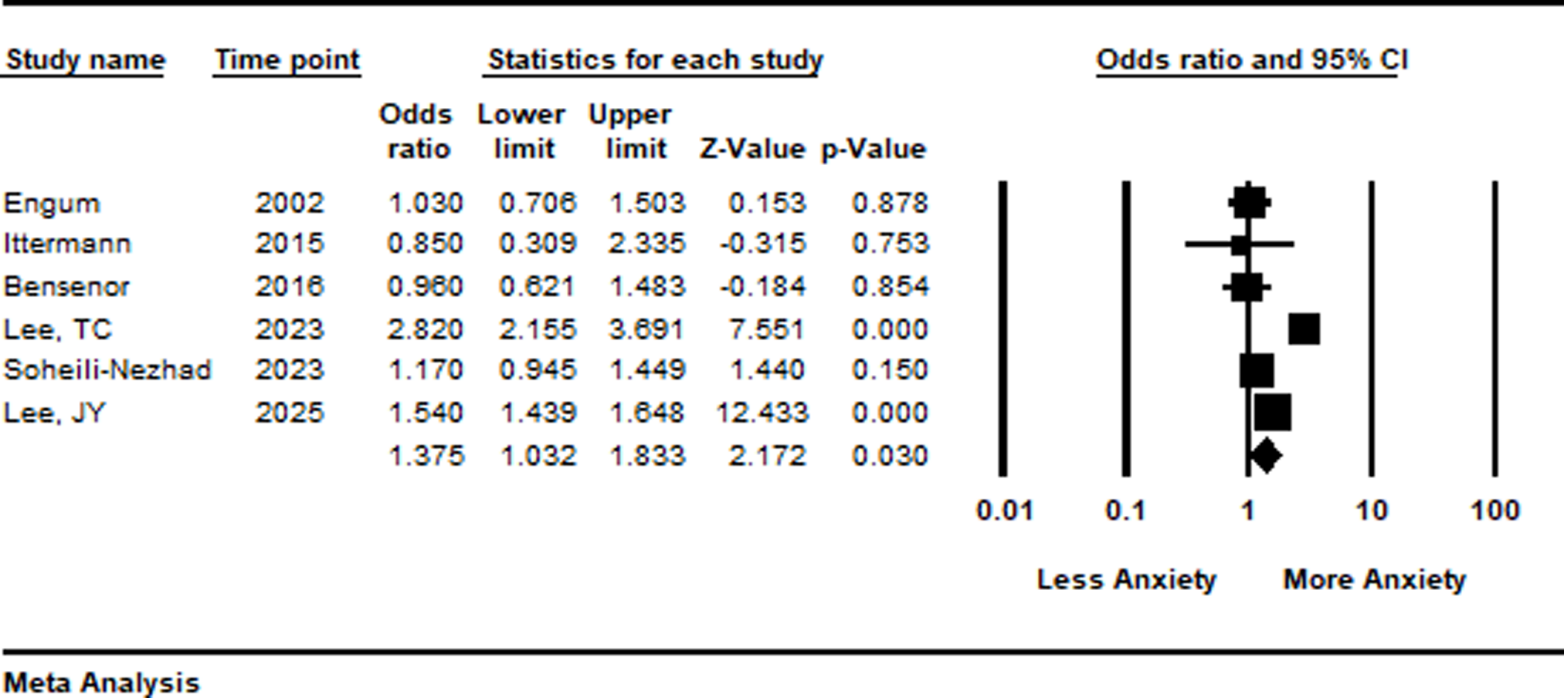
Forest plot: random effects meta-analysis of hyperthyroidism and clinical anxiety in population-based studies.

The pooled analysis of all included studies (*n* = 6) suggested a small but statistically significant association between hyperthyroidism and anxiety (OR 1.38, 95% CI [1.03–1.83]), albeit with substantial heterogeneity (*I*^2^ = 86%). In the sensitivity analysis using the Paule & Mandel estimator with Knapp–Hartung adjustment, the association was no longer statistically significant (OR 1.35, 95% CI [0.86–2.14]; *I*^2^ = 91%). Likewise, exclusion of the study by Lee et al. (2023) attenuated the pooled estimate and rendered it non-significant (OR 1.2, 95% CI [0.94–1.52]; *I*^2^ = 72%). Further subgroup analyses also showed no statistically significant associations for either clinical hyperthyroidism (OR 1.26, 95% CI [0.98–1.60]; *I*^2^ = 72%) or subclinical hyperthyroidism (OR 1.09, 95% CI [0.86–1.37]; *I*^2^ = 0%). Overall, the statistically significant result of the main analysis was not supported by the sensitivity analyses (Table 2, Fig. 3).

In the study by Roberts et al. (2006), not included for its continuous endpoint, participants with subclinical hyperthyroidism, on average, scored higher on the HADS-Anxiety scale (Mean: 5.44 [95% CI [4.69–6.18]]) compared to those with overt hyperthyroidism (Mean: 3.43 [2.18-4.67]) or to euthyroid participants (Mean: 4.8 [4.62–5.03]). Notably, the upper bounds of all 95% confidence intervals remained well below the cut-off for clinically significant anxiety symptoms.

### Thyroid autoimmunity

#### TPO+

The meta-analysis demonstrated no statistically significant association between the presence of TPO antibodies and anxiety disorders OR 1.47 (0.73–2.94), with a moderate level of heterogeneity (*I*^2^ = 65%).

#### TRAb+

Lee et al. (2023) examined a cohort of 398 patients with thyroid eye disease (TED), along with 1,592 matched controls, and found a significant association with anxiety disorders, reporting an OR of 2.82 (2.16–3.70).

### Serum thyroid-stimulating hormone level

Studies on TSH levels showed no clear association of serum TSH levels with clinical anxiety. The studies differed considerably in the way this relationship was measured:

In a sample of 9,319 men and 17,694 women, Panicker et al. (2009) calculated a lower chance of clinically relevant anxiety symptoms according to HADS-A with any mU/L increase in serum TSH, suggesting fewer anxiety with more thyroid stimulation.

Samuels et al. (2016) found no statistically significant relationship between clinically relevant symptoms on the Goldberg Anxiety Scale and an increase in TSH (OR 0.89 [0.72–1.1]).

Shrestha et al. (2016) showed a small and statistically non-significant negative correlation between serum-TSH and State-Trait Anxiety Inventory score (Spearman’s r: −0.07) in a sample of 130 individuals.

### Serum thyroxine level (fT4)

Samuels et al. (2016) reported that the probability of achieving clinically relevant symptoms in the Goldberg Anxiety Scale was not associated with an increase or a decrease in serum-FT4 (OR 1.05 [0.84–4.06]).

Similarly, Shrestha et al., (2016) reported a small and statistically non-significant negative correlation between STAI and serum FT4 (Spearman’s r: −0.05).

## Discussion

Based on the data collected in this review and meta-analysis, the current evidence does not support a clear and consistent association between thyroid and anxiety disorders. We found small positive associations with confidence intervals straddling unity and including medium-range effects as well as weak protective effects of thyroid pathology. The available evidence remains inconclusive including both clinical and subclinical hypothyroidism, as well as for thyroid-stimulating hormone levels and thyroid peroxidase antibodies. Similarly, the evidence was inconsistent for hyperthyroidism, encompassing both clinical and subclinical forms, free thyroxine levels and thyrotropin Receptor Antibodies.

### Hypothyroidism

The summary effect estimate (OR 1.13; 95% CI [0.77–1.65]; *I*^2^ = 86%), indicated a small and statistically non-significant, heterogeneous association. While two large-scale studies demonstrated a statistically significant negative association (*i.e.*, more hypothyroidism–fewer anxiety disorders) (Engum et al., 2002; Benseñor et al., 2016), this pattern was not supported in other studies.

Some continuous data accord well with these negative findings, showing that higher serum TSH levels were not associated with more anxiety—if anything, the data point to an inverse relationship (Shrestha et al., 2016; Samuels et al., 2016; Panicker et al., 2009; Roberts et al., 2006). The clinical relevance of these results, however, remains doubtful. For example, Roberts et al. (2006) estimated that a 50 mU/L increase in TSH would be required to lower the HADS-A score by a single point—an unlikely change in physiological terms. At the same time, Panicker et al. (2009) reported an odds ratio (OR) of 0.77 for clinically relevant anxiety symptoms per one mU/L increase in TSH. Based on this, a theoretical increase of three mU/L would correspond to an OR of approximately 0.46. This calculation—assuming a linear relationship, which may not necessarily be present—suggests that even moderate elevations in TSH could be associated with a substantially lower likelihood of anxiety symptoms.

One possible explanation is that hypothyroid symptoms, such as bradycardia or fatigue, may attenuate physiological or psychological expressions of anxiety. Yet this remains speculative, and other studies found either no effect or a positive association. The findings of Ittermann et al. (2015) illustrate the complexity of current evidence: while a positive association was found between clinically diagnosed hypothyroidism and anxiety, no such association emerged when only elevated—but still normal—TSH levels were considered. Soheili-Nezhad et al. (2023) found a mild but significant association between hypothyroidism and any diagnosis of anxiety disorder, observed only when thyroid dysfunction was self-reported and not confirmed when diagnoses were based on ICD-coded hospital data. This discrepancy may suggest recall bias, misclassification, or differences in illness perception. The lack of consistency across studies may be explained by variations in population characteristics, diagnostic thresholds, or study design. We excluded one population-based study (Thvilum et al., 2014) reporting a positive association between hypothyroidism and anxiolytic use for lack of diagnostic validity because anxiolytics are used for a wide variety of symptoms or conditions beyond anxiety, including depression, insomnia, anesthesia, or dependence.

### Hyperthyroidism

The summary effect estimate (OR 1.38, 95% CI [1.03–1.83]; *I*^2^ = 86%) suggested a small association, although statistical significance depended on the analytical method applied. Using the DerSimonian & Laird tau estimator (DL)—a standard approach in random-effects meta-analysis—a small statistically significant effect was observed. However, this result should be interpreted with caution. DL is known to underestimate between-study variance, particularly when the number of studies is small and substantial heterogeneity is present, thereby increasing the risk of false-positive findings.

Moreover, some of the studies contributing to the positive association were subject to important limitations. For example, Soheili-Nezhad et al. (2023)—similar to findings on hypothyroidism—reported a significant association only when thyroid disorders were self-reported, but not when based on hospital or registry data, suggesting potential reporting bias or misclassification. Likewise, Lee et al. (2023) reported a strong association between thyroid eye disease and anxiety symptoms. This study was included in the meta-analysis on hyperthyroidism because TED is caused by Graves’ disease in the vast majority of cases (Burch et al., 2022). However, TED frequently involves disfiguring proptosis and visual impairment, which may contribute to psychological distress beyond endocrine dysfunction. Indeed, patients with moderate to severe TED—particularly when disfigurement is prominent—experience increased emotional distress even when euthyroid (Farid et al., 2005; Kahaly et al., 2005; Park et al., 2004), with additional evidence linking altered self-image, social withdrawal, and reduced confidence to visible facial changes (Clarke et al., 2003; Jensen & Harder, 2011; Wickwar et al., 2015). While recent reviews support these observations, they also emphasize that the specific contribution of disfigurement to anxiety and depression remains insufficiently understood (Al-Badri et al., 2024). In a leave-one-out analysis excluding the study by Lee et al. (2023), the pooled effect estimate was markedly attenuated (OR 1.2, 95% CI [0.94–1.52]) and no longer statistically significant, indicating a substantial influence of this single study on the overall result.

We excluded one population-based study reporting a positive association between hyperthyroidism and anxiolytic use due to limited diagnostic validity, in line with our approach in the hypothyroidism analysis, as anxiolytics are prescribed for a wide range of indications beyond anxiety (Brandt et al., 2014).

In the sensitivity analysis using the Paule & Mandel Method—recommended as an alternative to the DerSimonian & Laird Method and capable of providing more stable estimates in the presence of heterogeneity—no statistically significant effect was observed. Consistent with this overall lack of robustness, three large studies found no association (Engum et al., 2002; Ittermann et al., 2015; Benseñor et al., 2016). This is further supported by continuous data from population-based studies; for example, Roberts et al. (2006) found no relationship between serum FT4 levels and anxiety scores on the HADS-A scale.

### Autoimmunity—TPO Ab positivity

With an odds ratio of 1.47 [0.73–2.94] across three studies and a wide prediction interval TPO Ab positivity fits well into the generally inconclusive findings and is in contrast to results reported by Siegmann et al. (2018), who found a statistically significant association: OR 2.3 [1.40–3.85]. Unfortunately, the authors did not differentiate between types of thyroid dysfunction and treated all hypothyroidism as indicative of autoimmunity. Also, the inclusion of case-control studies increases the risk of arriving at odds ratios unrepresentative of the population level (Baethge, 2018).

The study concerning TPO-Abs with the largest sample size (Engum et al., 2005) found no association, while smaller studies (Carta et al., 2004) tended to report more pronounced effects, albeit without statistical significance. The results are consistent with those of Jensen et al. (Jensen et al., 2022), who also found no association between TPO antibody positivity and antidepressant use—a proxy outcome for affective disorders.

Two large-scale genome-wide association studies (Soheili-Nezhad et al., 2023; Zhou & Zhu, 2024) suggest a shared immunogenetic disposition of hypothyroidism and anxiety symptoms. However, a recent Mendelian randomization study found no evidence for a causal link between anxiety disorders and autoimmune thyroiditis in either direction (Su et al., 2025), indicating that the observed associations may reflect non-causal genetic overlap rather than a direct causal relationship.

### Interpretation of non-significant findings

The lack of statistically significant associations may be attributable to several factors. While thyroid dysfunctions can induce pronounced autonomic symptoms—such as tachycardia, sweating, tremor, or inner agitation—these physiological responses are clearly distinguishable from the diagnostic core features of anxiety disorders. Clinically relevant anxiety is defined by persistent cognitive–emotional fears, anticipatory anxiety, avoidance behavior, and substantial functional impairment. Increased physiological arousal alone therefore does not necessarily translate into an anxiety disorder, even when such symptoms are subjectively perceived as “anxiety-like”. This interpretation is consistent with findings from the largest available studies, which assessed thyroid function parameters (*e.g.*, TSH, fT4, fT3, TPO antibodies) alongside anxiety disorders using validated diagnostic approaches and reported no evidence of an association. In contrast, smaller studies tended to yield larger effect estimates—a pattern more suggestive of random variation or methodological bias than of a robust underlying relationship.

The associations observed in several case–control studies may be more plausibly explained by selection processes and burden-related biases. Clinical samples disproportionately include individuals with more severe disease courses, and it is well established that the general burden of chronic illness can exacerbate psychological symptoms, including anxiety (Liu et al., 2021; Abbas et al., 2025; Bobo et al., 2022). As diagnostic and therapeutic complexity increases, so too may psychological strain (Gebreyohannes et al., 2023). Elevated anxiety symptoms in clinical thyroid populations may therefore reflect illness-related burden rather than direct pathophysiological effects of thyroid hormone dysregulation.

At the same time, the present findings do not permit a definitive exclusion of an association between thyroid dysfunction and anxiety disorders. Several methodological limitations and additional factors may contribute to the absence of statistically significant results.

First, the meta-analysis is based on a limited number of eligible studies that vary considerably with respect to methodological quality, study design, definitions of thyroid pathology, anxiety assessment, and study populations. In particular, diagnostic approaches to anxiety disorders differed substantially across studies, ranging from clinical interviews and registry-based diagnoses to questionnaires and self-report measures, each with differing susceptibility to misclassification.

Second, both thyroid dysfunctions and anxiety disorders may follow episodic courses and do not necessarily co-occur temporally. As most included studies—apart from two cohort studies—were cross-sectional in design, potential temporal associations may have remained undetected and manifested as null findings.

Third, hyperthyroidism is a relatively rare condition. Even large population-based datasets therefore contain only small numbers of affected individuals, resulting in wide confidence intervals and unstable effect estimates. This increases the likelihood of non-significant or inconsistent findings, even in the presence of a true association.

### Comparison with depression

The present results are part of a project which resulted in meta-analyses of population-based studies on the relationship between hypo- as well as hyperthyroidism and depression (Bode et al., 2022; Bode et al., 2021). Of note, there are more studies on clinical depression than on anxiety, which is surprising, as anxiety disorder is highly relevant to society and even more frequent than depression (Baxter et al., 2013; Baxter et al., 2014). Our meta-analyses revealed a small association between hypothyroidism and depression (OR 1.30 [1.08-1.57]), and a statistically not significant association for autoimmunity (OR 1.24 [0.89-1.74]). The association between hyperthyroidism and depression was stronger with an OR of 1.67 [1.49–1.87]. In a recent analysis of UK Biobank data, Fan and co-workers (Fan et al., 2024) took a different view: Their starting point was not a thyroid pathology but a positive screening result for depression/anxiety in the Patient Health Questionnaire-4 (PHQ-4). Among positively screened participants the probability of developing any thyroid disorder—hyper-, hypothyroidism and autoimmunity, as determined by hospital diagnoses—was increased when compared to non-anxious participants during 13 years of follow-up. Although, the results have not been adjusted for multiplicity, crude hazard ratios did not exceed 1.9 and became smaller when the model had been adjusted for some possible confounders. It is also unclear how many participants who screened positive truly had a clinical diagnosis of anxiety disorders and to what degree detection bias among patients treated for depression or anxiety may have influenced the findings. If the results hold, however, they would fit in the order of magnitude of the association that emerged from the meta-analyses on depression (Bode et al., 2022; Bode et al., 2021).

## Limitations

As mentioned before, key limitations of this review include a small number of studies, varying methodological quality, and substantial heterogeneity across designs, populations, and outcome definitions. Consequently, our results are preliminary. In addition, Egger’s test or trim-and-fill analyses were not performed because the number of studies available per outcome was far below the recommended minimum for these procedures, which would have rendered the results unreliable due to insufficient statistical power (Rao et al., 2017). Also, two studies were population-based only in a wider sense (Benseñor et al., 2016; Roberts et al., 2006) but the samples seem acceptable approximations of the population, particularly because it is difficult to see why relative risks should deviate substantially.

Another possible confounder is levothyroxine treatment: Two studies Engum et al. (2002) and Panicker et al. (2009) reported a higher prevalence of anxiety symptoms in LT4-treated individuals relative to unmedicated participants. Similar patterns were also observed in larger cohorts: Jensen et al. (2022) found increased antidepressant use only among LT4 users. Similarly, a study of 70,000 patients with type 1 diabetes reported no general association between autoimmune thyroid disease and antidepressant use, but observed a significant increase exclusively in LT4-treated men (Eckert et al., 2021). In contrast, Ittermann et al. (2015) reported a higher prevalence of anxiety diagnoses in untreated rather than in treated hypothyroid individuals.

## Conclusion

We did not find consistent evidence of an association between clinical and subclinical thyroid pathology and anxiety disorders in population-based studies. Future large-scale prospective studies with standardized diagnostic assessments are required to reliably clarify the relationship between thyroid and anxiety disorders. Given the current state of evidence, consistent, guideline-based treatment is warranted for both thyroid disorders and anxiety disorders but one should not rely on carry-over effects of treating thyroid pathology on anxiety disorders; however, carry-over effects of treating thyroid pathology on anxiety outcomes should not be assumed.

## Supplemental Information

10.7717/peerj.21446/supp-1 Supplemental Information 1 Search history

10.7717/peerj.21446/supp-2 Supplemental Information 2 Raw data

10.7717/peerj.21446/supp-3 Supplemental Information 3 Systematic Review and Meta-Analysis Rationale

## Additional Information and Declarations

### Data Availability

The following information was supplied regarding data availability: The raw data is available in the Supplemental Files.
