# Supplementary material for: Thyroid disorders, thyroid autoimmunity, and anxiety—a systematic review and meta-analysis of population-based studies
Source: PeerJ. 2026 Aug 4;14:e21446. doi: 10.7717/peerj.21446 (PMC13450037; doi:10.7717/peerj.21446)
Supplement: Supplementary file 1 [file peerj-14-21446-s001.docx]

Search Criteria

The database search was performed in three separate databases: Medline (via PubMed), PsycINFO (via EBSCOhost) and Embase. Since databases require a different syntax, different search requests were created.

PubMed:

(((Hashimoto*[Text Word] OR "Graves Disease"[Text Word] OR AIT[Text

Word] OR Basedow[Text Word] OR "Ord* Disease"[Text Word] OR

thyroid*[Text Word] OR hypothyroid*[Text Word] OR hyperthyroid*[Text

Word] OR Hypothyroidism[MeSH Terms] OR Hyperthyroidism[MeSH Terms]

OR "Thyroiditis, Autoimmune"[MeSH Terms] OR "Hashimoto Disease"[MeSH

Terms] OR "Graves Disease"[MeSH Terms] OR Thyroid diseases[MeSH

Terms] OR Thyroiditis[MeSH Terms] OR "Thyroiditis, subacute"[MeSH

Terms] OR "Antibodies, Thyroid-Stimulating"[MeSH Terms]))

AND

(Anx*[Text Word] OR Fear[Text Word] OR GAD-7[Text Word] OR

Phob*[Text Word] OR Anxiety[Text Word] OR Angst[Text Word] OR

nervousness[Text Word] OR Agitation[Text Word] OR Panic[Text Word]

OR "Anxiety Disorders"[MeSH Terms] OR "Manifest Anxiety Scale"[MeSH

Terms] OR "Test Anxiety Scale"[MeSH Terms] OR "Patient Health

Questionnaire"[MeSH Terms] OR Fear[MeSH Terms] OR "Anti-Anxiety

Agents"[MeSH Terms] OR Anxiety[MeSH Terms]))

AND

(epidemiolog*[Text Word] OR cohort[Text Word] OR random*[Text Word]

OR cross-sectional[Text Word] OR population[Text Word] OR

prospective[Text Word] OR "Epidemiologic Studies"[MeSH Terms] OR

"Cohort Studies"[MeSH Terms] OR "Cross-Sectional Studies"[MeSH

Terms] OR epidemiology[MeSH Terms] OR "Anxiety

disorders/epidemiology"[MeSH Terms] OR

"Hypothyroidism/epidemiology"[MeSH Terms] OR

"Hyperthyroidism/epidemiology"[MeSH Terms] OR "Mental

disorders/epidemiology"[MeSH Terms] OR "Metabolic

diseases/epidemiology"[MeSH Terms] OR "Anxiety/epidemiology"[MeSH Terms])

EMBASE/EBSCOhost:

Hashimoto* OR "Graves Disease" OR AIT OR Basedow OR "Ord* Disease" OR thyroid* OR hypothyroid* OR hyperthyroid*

AND

Anx* OR Fear OR GAD-7 OR Phob* OR Anxiety OR Angst OR nervous* OR Agitation OR Panic OR "HAM-A"

AND

epidemiolog* OR cohort OR random* OR cross-sectional OR population OR prospective
