# Supplementary material for: Thyroid disorders, thyroid autoimmunity, and anxiety—a systematic review and meta-analysis of population-based studies
Source: PeerJ. 2026 Aug 4;14:e21446. doi: 10.7717/peerj.21446 (PMC13450037; doi:10.7717/peerj.21446)
Supplement: Supplementary file 2 [file peerj-14-21446-s003.docx]

**Systematic Review and Meta-Analysis Rationale:**

Anxiety disorders are the most prevalent psychiatric conditions worldwide, and thyroid dysfunction — particularly hypothyroidism and hyperthyroidism — has repeatedly been linked to anxiety symptoms. This association has often been replicated in clinical case-control studies. However, such studies are prone to selection bias because they rely on clinical samples that may not be representative of the general population. In contrast, population-based studies, which are less prone to bias, have produced highly inconsistent and sometimes contradictory findings regarding the association between thyroid dysfunction and anxiety.

To our knowledge, only one previous meta-analysis (Siegmann et al., 2018) has addressed this association, but it primarily relied on small case-control studies and did not focus on population-based data. No meta-analysis has, to our knowledge, specifically examined this association based solely on population-based studies, although such studies are better suited to estimate the true association between thyroid disorders and anxiety in the general population.

The present systematic review and meta-analysis addresses this gap by synthesizing evidence exclusively from population-based cohort and cross-sectional studies. This approach minimizes the risk of selection bias and allows for a more accurate estimation of the association between thyroid dysfunction and anxiety disorders. In addition, this work differentiates between overt and subclinical forms and includes analyses on thyroid autoimmunity, offering new insights beyond previous reviews.
